# Aerobic Exercise Training Prevents Insulin Resistance and Hepatic Lipid Accumulation in LDL Receptor Knockout Mice Chronically Fed a Low-Sodium Diet

**DOI:** 10.3390/nu13072174

**Published:** 2021-06-24

**Authors:** Guilherme da Silva Ferreira, Ana Paula Garcia Bochi, Paula Ramos Pinto, Vanessa Del Bianco, Letícia Gomes Rodrigues, Mychel Raony Paiva Teixeira Morais, Edna Regina Nakandakare, Ubiratan Fabres Machado, Sergio Catanozi, Marisa Passarelli

**Affiliations:** 1 Laboratorio de Lipides (LIM-10), Hospital das Clinicas (HCFMUSP) da Faculdade de Medicina da Universidade de São Paulo, São Paulo 01246-903, Brazil; ferreira.gui@usp.br (G.d.S.F.); bochianapaula@gmail.com (A.P.G.B.); paularamos@usp.br (P.R.P.); vdelbianco84@gmail.com (V.D.B.); leticia_g_rodrigues@hotmail.com (L.G.R.); enakonda@usp.br (E.R.N.); catanozi@usp.br (S.C.); 2 Department of Cell and Development Biology, Institute of Biomedical Sciences, University of São Paulo, São Paulo 05508-000, Brazil; raony@usp.br; 3 Department of Physiology and Biophysics, Institute of Biomedical Sciences, University of São Paulo, São Paulo 05508-000, Brazil; ubiratan@icb.usp.br; 4 Programa de Pós-Graduação em Medicina, Universidade Nove de Julho, São Paulo 01525-000, Brazil

**Keywords:** sodium restriction, aerobic exercise, insulin resistance, dyslipidemia

## Abstract

Background: A low-sodium (LS) diet reduces blood pressure, contributing to the prevention of cardiovascular diseases. However, intense dietary sodium restriction impairs insulin sensitivity and worsens lipid profile. Considering the benefits of aerobic exercise training (AET), the effect of LS diet and AET in hepatic lipid content and gene expression was investigated in LDL receptor knockout (LDLr-KO) mice. Methods: Twelve-week-old male LDLr-KO mice fed a normal sodium (NS) or LS diet were kept sedentary (S) or trained (T) for 90 days. Body mass, plasma lipids, insulin tolerance testing, hepatic triglyceride (TG) content, gene expression, and citrate synthase (CS) activity were determined. Results were compared by 2-way ANOVA and Tukey’s post-test. Results: Compared to NS, LS increased body mass and plasma TG, and impaired insulin sensitivity, which was prevented by AET. The LS-S group, but not the LS-T group, presented greater hepatic TG than the NS-S group. The LS diet increased the expression of genes related to insulin resistance (*ApocIII*, *G6pc*, *Pck1*) and reduced those involved in oxidative capacity (*Prkaa1*, *Prkaa2*, *Ppara*, *Lipe*) and lipoprotein assembly (*Mttp*). Conclusion: AET prevented the LS-diet-induced TG accumulation in the liver by improving insulin sensitivity and the expression of insulin-regulated genes and oxidative capacity.

## 1. Introduction

Dietary sodium restriction reduces blood pressure (BP) contributing to preventing cardiovascular diseases (CVD). Less than 2 g of sodium/day (corresponding to 5 g of sodium chloride) is recommended by the World Health Organization as a public health strategy to deal with cardiovascular morbidity and mortality [1]. However, recent observational studies have indicated a U- or J-shaped curve for CVD or mortality related to daily sodium consumption below 3 g or above 7 g [2]. Long-term intensive sodium restriction in the diet activates the renin-angiotensin-aldosterone system (RAAS), and the sympathetic nervous system favoring insulin resistance. Those events contribute to the increase in plasma lipids, especially triglycerides (TG) and free fatty acids (FFA), enhanced lipid infiltration in the arterial wall, and glycoxidative stress [3]. It was recently demonstrated that LDL receptor knockout (LDLr-KO) mice chronically fed a low-sodium (LS) diet present many alterations in skeletal muscle lipidomics, namely glycerophospholipid and fatty acid species that relate to the reduced insulin sensitivity observed in those animals [4].

There are limited data on the influence of an LS diet in lipid metabolism in the liver, a central organ for lipid and glucose homeostasis. Prada et al. (2005) observed a reduced insulin sensitivity in Wistar rats fed an LS diet that was attributed to the activation of c-Jun N terminal kinase (JNK) proteins and serine phosphorylation of the insulin receptor substrate 1 (IRS-1 ser307) [5]. Xavier et al. (2003) reported greater de novo lipogenesis, although without an increase in TG content in the liver [6]. It was recently demonstrated that LS intake increased hepatic diacylglycerol, esterified cholesterol, and inflammation in mice fed with a high-fat diet for 12 weeks. However, high sodium diet-fed animals were protected against steatosis induced by a high-fat or a choline/methionine deficient diet, which was ascribed to the reduced activation of the mineralocorticoid receptor helping to reduce lipogenesis markers in the liver [7].

Regular exercise improves insulin sensitivity and lipid metabolism, reducing plasma TG and small-dense low-density lipoprotein (LDL). Moreover, it increases cholesterol in high-density lipoprotein (HDL) and ameliorates the reverse cholesterol transport and antioxidant defenses in the arterial wall [8,9]. Aerobic exercise training (AET) favors fatty acid oxidation, limits hepatic TG accumulation, and impairs the detrimental actions of fatty acid derivatives in the insulin receptor signaling cascade [10,11].

The present study investigated the effect of an intensive and chronically administered LS diet and AET in the modulation of lipid content and expression of genes related to lipid metabolism and insulin sensitivity in the liver of LDLr-KO mice.

## 2. Materials and Methods

### 2.1. Animals

C57BL/6J background homozygous LDLr-KO mice (Jackson Laboratory, Bar Harbor, ME, USA) were housed in a conventional animal facility at 22 °C ± 2, under a 12 h light/dark cycle with free access to commercial chow (Nuvilab CR1-Nuvital Nutrients, Colombo, PR, Brazil) and drinking water. 

### 2.2. Experimental Protocol

The experimental protocol was approved by the Animal Care and Research Advisory Committee of the Faculdade de Medicina da Universidade de Sao Paulo (CEUA # 1210/2019) and it was conducted according to the U.S. National Institutes of Health Guide for the Care and Use of Laboratory Animals. Twelve-week-old mice were randomly divided into four groups in a factorial design 2 × 2 according to sodium consumption and AET: 1—normal sodium (NS) diet plus sedentary (NS-S; *n* = 14); 2—NS diet plus trained (NS-T; *n* = 13); 3—LS diet plus sedentary (LS-S; *n* = 13); and 4—LS diet plus trained (LS-T; *n* = 13). The intervention protocol lasted 90 days.

Maximum exercise capacity, body mass, systolic blood pressure (SBP), diastolic blood pressure (DBP), heart rate, hematocrit, plasma total cholesterol (TC), **TG**, and glucose were determined before and after the intervention. In addition, 24 h urinary sodium excretion, insulin tolerance testing (kITT), and plasma lipoprotein profile were determined only at the end of the intervention. 

Mice were euthanized with an overdose of sodium thiopental (Thiopentax^®^; 150 mg/kg body mass) and the liver was excised. A fragment was snap-frozen in liquid nitrogen and stored at −80 °C for further analysis of lipid content, and other fragments were stored for 24 h at −20 °C and then at −80 °C in RNAlater^TM^ Stabilization Solution (Thermofisher Scientific, Waltham, MA, USA) for measuring gene expression or immediately processed for histological analysis. 

### 2.3. Diet and Aerobic Exercise Training

Low-sodium (0.06% Na^+^; Envigo Teklad Diets; Indianapolis, IN, USA, TD 92141) and NS diets (0.5% Na; Envigo Tekald, TD92140) were similar in energy and nutrients (g/100 g): casein (28.7), sucrose (31.3), cornstarch (20.0), soybean oil (6.0), cellulose (9.79), vitamin mix (Teklad—1.0), and ethoxyquin. In the LS diet, sodium chloride was replaced for cellulose but its amount was sufficient for normal mouse growth rate. Food consumption (g/day/number of animals in cage) was measured weekly by difference from given and uneaten diet mass. For this analysis, each group of animals was considered one experimental unity.

The AET was performed on a treadmill 5 days/week, at 15 m/min, for 60 min. In the first week, mice were acclimated at a speed of 12 m/min with a gradual increase in time per session (10 min) from 30 to 60 min. Throughout the protocol, 10% of the trained animals were excluded by the incapacity of running.

### 2.4. Treadmill Exercise Test

A progressive treadmill exercise test until exhaustion was performed after 3 sessions of exercise (15 m/min, 10 min) as previously described [12]. The speed started at 9 m/min and 0% grade with 3 m/min at every 3 min being added until the complete inability to run, even after constantly stimulus. The time to exhaustion, the maximum time that the animal was able to run, was used as the parameter of physical conditioning.

### 2.5. Blood Glucose, Hematocrit, Plasma Lipids, Blood Pressure, and Urinary Sodium Excretion

Blood from the tail vein was collected (~200 µL) in heparinized capillary tubes after a 12 h overnight fasting period. Hematocrit was quantified by the microhematocrit technique. Plasma TG and TC were determined by enzymatic colorimetric kits (Labtest, Lagoa Santa, MG, Brazil) and glycemia by an Accu-Chek^®^ Performa glucometer. SBP and DBP were assessed in conscious animals by non-invasive photoplethysmography with a computerized tail-cuff system (Visitech Systems, model BP-2000 Blood Pressure Analysis SystemTM—Apex, NC, USA). Animals were preconditioned to the method before final analyses and mean values were obtained after 8 consecutive measurements. Twenty-four hour urinary collection was accomplished in individual metabolic cages and utilized for the determination of urinary sodium in an FC 280 flame spectrophotometer (CELM; São Paulo, SP, Brazil).

### 2.6. Lipoprotein Profile

Plasma lipoprotein profile was determined by fast protein liquid chromatography (FPLC) on an HR 10/30 Superose 6 column with a constant flow of 0.5 mL/min of Tris-buffered saline, pH 7.2, utilizing 100 μL of plasma. The amount of TC and TG associated with very-low-density lipoprotein (VLDL), low-density lipoprotein (LDL), and high-density lipoprotein (HDL) was determined by enzymatic techniques.

### 2.7. Insulin Tolerance Test

The insulin tolerance test (kITT) was performed in the 10th week after 4 h fasting. Glycemia was assessed (Accu-Chek^®^ Performa) at basal, 10, 20, and 30 min after intraperitoneal injection of regular insulin (1 U/kg—Humulin, Eli Lilly). The glucose decay rate was determined by linear regression. 

### 2.8. Hepatic Lipid Content

Lipid extraction was performed according to Carr et al. (1993) [13]. Briefly, 100 mg of liver tissue was macerated in a solution of chloroform:methanol (2:1, *v*:*v*) and samples were maintained overnight at –20 °C. The organic phase was separated adding aqueous solution containing 0.05% H_2_SO_4_ following centrifugation (1690× *g*). The solvent was dried (Genivac Standard [EZ-2], Ipswich, Suffolk, England), added with 1 mL Triton X-100 solution (0.5% in chloroform) and dried again (Genivac Standard [EZ-2], Ipswich, Suffolk, England). The sample was resuspended in 500 µL of water and heated at 37 °C for 15 min with agitation. TG and TC were determined by enzymatic colorimetric kits (Labtest, Lagoa Santa, MG, Brazil) in automated equipment (COBAS-MIRA—Roche Diagnostics, Indianapolis, IN, USA). The aqueous fraction was dried and diluted in 1 mL of 1N NaOH. Protein concentration was determined by the method of Lowry et al. (1951) [14]. TC and TG concentration was corrected by g of liver and g of protein.

### 2.9. Glycogen Quantification 

Liver sections from the median lobe (~3.0 µm) included in paraffin were stained with Periodic Acid-Schiff (PAS). Histological sections were photographed around centrilobular veins, with a 20x objective, using an Olympus DP72 optical microscope equipped with a digital camera. For each animal, 10 microscopic fields of at least 2 cuts were quantified, using the Image J program (National Institutes of Health, USA). Results are presented as percentage of stained area.

### 2.10. Real-Time Quantitative PCR (RT-qPCR)

A fragment of the middle lobe (~30 mg) was macerated using 600 µL of lysis buffer (RLT, Qiagen, Hilden, North Rhine-Westphalia, Germany) with 1% beta-mercaptoethanol. RNA extraction and purification were performed using the RNeasy Mini Kit (Qiagen, Hilden, North Rhine-Westphalia, Germany). The RNA quantity and quality were performed by the Lab-on-a-Chip Approach of Agilent using a 2100 Bioanalyzer. cDNA was obtained from 1000 ng of total RNA using a commercial High-Capacity RNA-to-cDNA kit (Applied Biosystems, Thermofisher Scientific, Waltham, MA, USA). The volume of cDNA was then diluted 10x in endonuclease-free water and stored at −20 °C. mRNA expression was measured by real-time quantitative polymerase chain reaction (RT-qPCR) using TaqMan probes (Applied Biosystems, Thermofisher Scientific, Waltham, MA, USA) in Step One Plus Real-Time qPCR System (Applied Biosystems, Thermofisher Scientific, Waltham, MA, USA): *Acaca*, acetyl-Coenzyme A carboxylase alpha (Mm01304257_m1); *Ager*, advanced glycosylation end product-specific receptor (Mm00545815_m1); *Apoc2*, Apolipoprotein C-II (Mm00437571_m1); *Apoc3*, apolipoprotein C-III (Mm00445670_m1); *Ddost*, dolichyl-di-phosphooligosaccharide-protein glycotransferase (Mm00492100_m1); *Fasn*, fatty acid synthase (Mm00662319_m1); *Foxa2*, forkhead box A2 (Mm00839704_mH); *Foxo1*, forkhead box O1 (Mm00490671_m1); *G6pc*, glucose-6-phosphatase, catalytic (Mm00839363_m1); *Il6*, Interleukin-6 (Mm00446190_m1); *Il10*, Interleukin-10 (Mm01288386_m1); *Lipe*, lipase, hormone sensitive (Mm00495359_m1); *Lrp1*, low density lipoprotein receptor-related protein 1(Mm00464608_m1); *Mttp*, microsomal triglyceride transfer protein (Mm00435015_m1); *Pck1*, phosphoenolpyruvate carboxykinase 1, cytosolic (Mm01247058_m1); *Plin1*, perilipin 1(Mm00558672_m1); *Ppara*, peroxisome proliferator activated receptor alpha (Mm00440939_m1); *Ppargc1a*, peroxisome proliferative activated receptor, gamma, coactivator 1 alpha (Mm01208835_m1); *Prkaa1* and *Prkaa2*, protein kinase, AMP-activated, alpha 1(Mm01296700_m1) and alpha 2 (Mm01264789_m1) catalytic subunit; *Rela*, v-rel reticuloendotheliosis viral oncogene homolog (Mm00501346_m1); *Slc2a2*, solute carrier family 2 (facilitated glucose transporter) member 2 (Mm00446229_m1); *Srebf1* and *Srebf2*, sterol regulatory element binding transcription factor 1 (Mm00550338_m1) and 2 (Mm01306292_m1). The relative quantification of gene expression was calculated by using the comparative cycle threshold (CT; 2^−ΔΔCT^) method [15]. Beta-actin (Mm02619580_g1) was chosen as the housekeeping gene after excluding other candidate genes (*Actb, Gapdh* [Mm99999915_g1], *Hprt* [Mm03024075_m1], *Rplp0* [Mm00725448_s1], *Ppib [Mm00478295_m1]*) after running a gene stability test (Normfinder, https://moma.dk/normfinder-software; accessed on 30 July 2019). 

### 2.11. Citrate Synthase Activity

The activity of the citrate synthase was evaluated in liver homogenates (32 µg of protein), by measuring the rate of 5-thio-2-nitrobenzoic acid (TNB) formation at 412 nm (#CS0720, Sigma-Aldrich, St. Louis, MO, USA). The kinetic assay was performed in a 96-well plate, with a final volume of 105 µL of the reaction mixture (30 mM acetyl-Coa, 5’5-Dithio-bis-(2-nitrobenzoic) acid), 10 mM, 10 mM oxaloacetate, 32 µg of liver supernatant protein) for 2 min.

### 2.12. Statistical Analysis

Statistical analysis was performed using the Minitab 19 software. Data normality was checked by the Kolmogorov–Smirnov test, non-normal variables were log- or Johnson-transformed before analysis of variance. Differences at the baseline were compared by one-way ANOVA; results after intervention were compared by 2-way ANOVA (generalized linear model) with fixed factors: diet and exercise. Tukey’s post-test was applied when appropriate. In order to verify pre- and post-effects, the analyses of exercise capacity and food consumption were running by repeated measures ANOVA (mixed model; random factor: animal and fixed factors: diet, exercise, and time). Correlations between variables were determined using Spearman’s correlation test. *p* < 0.05 was considered statistically significant. The statistical differences could be indicated by the effect of diet (NS vs. LS), exercise (S vs. T), or interaction. When interaction was reached, distinct letters represent statistical differences among all groups (*p* < 0.05). Data are presented as mean ± standard deviation (SD).

## 3. Results 

### 3.1. Efficiency of Interventions

**Figure 1:**
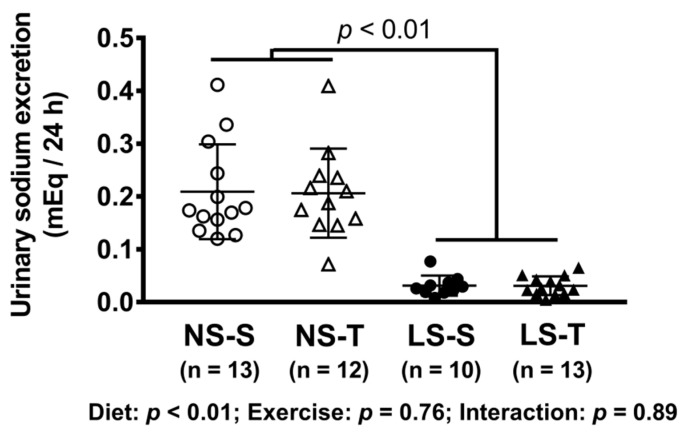
Urinary sodium excretion (mEq/24 h). Data presented as mean ± SD. Results compared by 2-way ANOVA (GLM; fixed factor: diet and exercise). *p* value shows difference between NS and LS groups. *p* exercise = 0.94; *p* diet*exercise = 0.94. NS = normal-sodium diet; LS = low-sodium diet; S = sedentary; T = trained.

**Figure 2:**
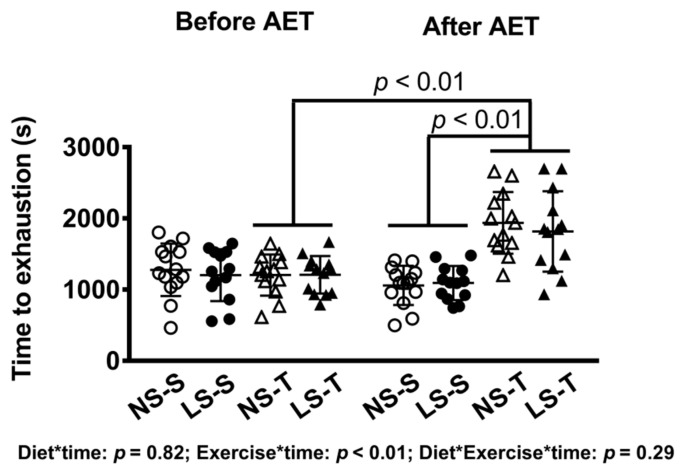
Time to exhaustion in treadmill exercise test before and after AET. Data presented as mean ± SD. Results compared by 3-way ANOVA (random factor: animal; fixed factor: time, diet and exercise) with Tukey’s post hoc test. *p* exercise*time < 0.01; *p* exercise*diet*time = 0.32. NS = normal-sodium diet; LS = low-sodium diet; S = sedentary; T = trained. NS-S *n* = 14; NS-T *n* = 13; LS-S *n* = 13; LS-T *n* = 13.

As expected, a diminished 24 h urinary sodium excretion was observed in the LS as compared to the NS group (Figure 1). In AET groups, a 60% improvement in the exercise capacity was observed over time and a 79% increment in aerobic capacity was observed in comparison to sedentary animals at the end of the training protocol (Figure 2). Food intake was similar among groups (data not shown). 

### 3.2. Metabolic Parameters

Body mass, TC, TG, blood glucose, hematocrit, SBP, and DBP were similar among groups at the beginning (Table 1). After intervention, the LS groups showed greater body mass, glycemia, and triglyceridemia compared to NS groups (Table 2). Biochemical data from NS-S and LS-S animals were presented elsewhere as a part of the evaluation of muscle lipidomics in those animals [4]. Unexpectedly, AET mice presented higher blood glucose compared to sedentary ones. Hematocrit, TC, SBP, and DBP were not altered by either diet or AET (Table 2). 

### 3.3. Insulin Tolerance Test (kITT) 

**Figure 3:**
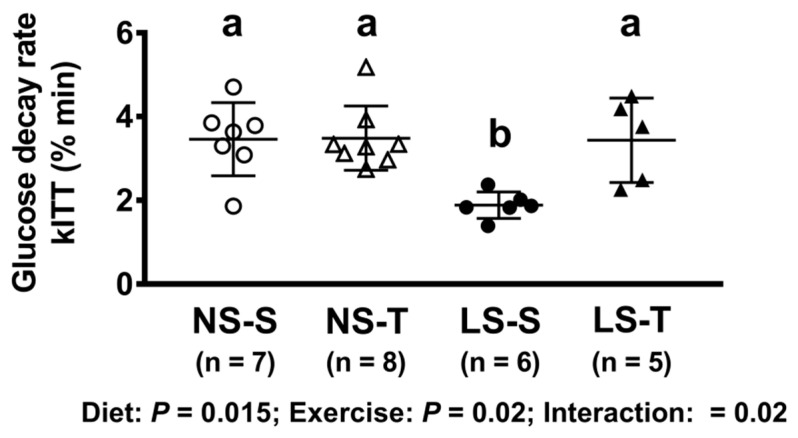
Glucose decay rate in insulin tolerance test (kITT, % min). Data presented as mean ± SD. Results compared by 2-way ANOVA (GLM; fixed factor: diet and exercise) with Tukey’s post hoc test. *p* diet = 0.02; *p* exercise = 0.02; *p* diet*exercise = 0.02. Distinct letters represent statistical differences among groups (*p* < 0.05). NS = normal-sodium diet; LS = low-sodium diet; S = sedentary; T = trained.

In the kITT, a reduced glucose decay rate was observed in the LS-S group as compared to others, as previously reported [4]; however, the impairment in the insulin sensitivity was prevented by AET (Figure 3). 

### 3.4. Plasma Lipoprotein Profile

**Figure 4:**
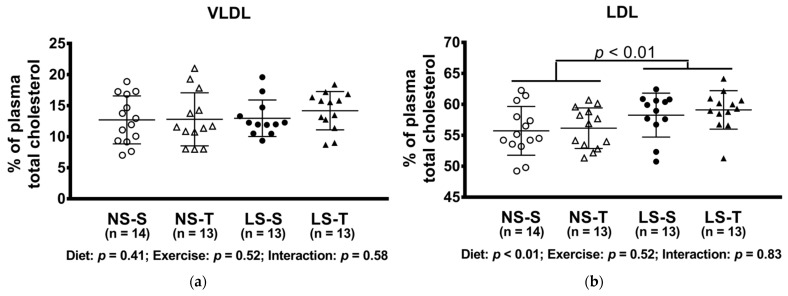

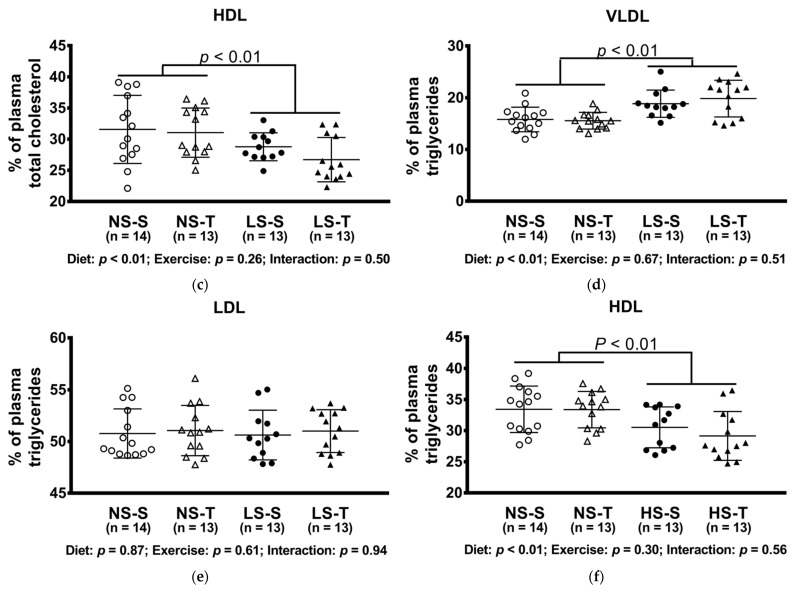
Percentual of plasma lipids in lipoproteins determined by FPLC. (**a**) VLDLc; (**b**) LDLc; (**c**) HDLc; (**d**) VLDLtg; (**e**) LDLtg; (**f**) HDLtg. Data presented as mean ± SD. Results compared by 2-way ANOVA (GLM; fixed factor: diet and exercise) with Tukey’s post hoc test. *p* values show differences between diets. NS = normal-sodium diet; LS = low-sodium diet; S = sedentary; T = trained.

The LS diet altered lipoprotein profile, shifting cholesterol to LDL and TG to VLDL particles, while both lipids were decreased in the HDL fraction (Figure 4). 

### 3.5. Triglyceride and Glycogen Content in the Liver

**Figure 5:**
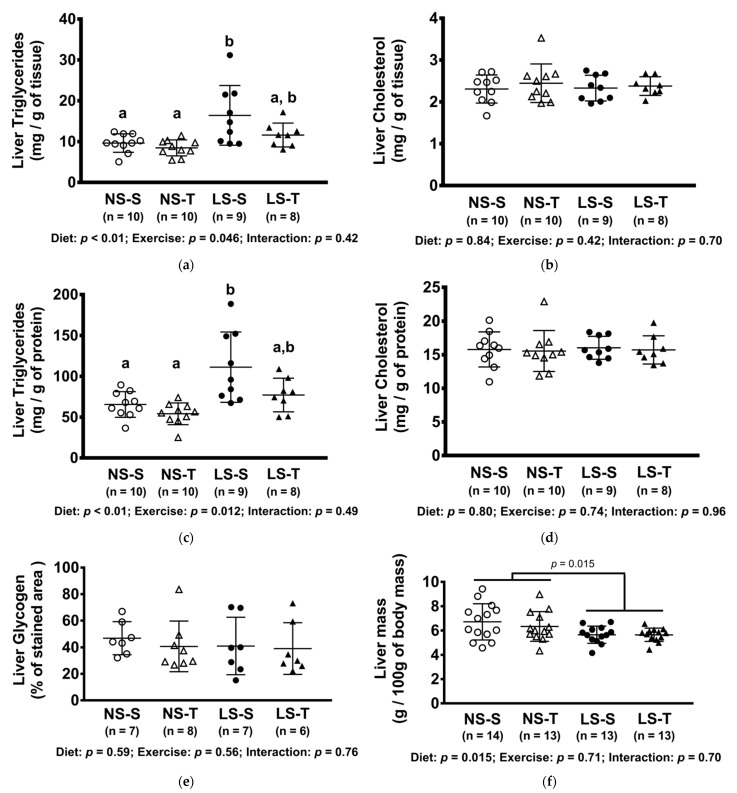
Concentration of hepatic lipids and glycogen, and liver mass. (**a**) Triglycerides (mg/g of tissue); (**b**) cholesterol (mg/of tissue); (**c**) triglycerides (mg/g of protein); (**d**) cholesterol (mg/g of protein); (**e**) glycogen (% of stained area); (**f**) liver mass (g/100 g of body mass). Data presented as mean ± SD. Results compared by 2-way ANOVA (GLM; fixed factor: diet and exercise) with Tukey’s post hoc test. Distinct letters represent statistical differences between groups (*p* < 0.05). NS = normal-sodium diet; LS = low-sodium diet; S = sedentary; T = trained.

**Figure 6:**
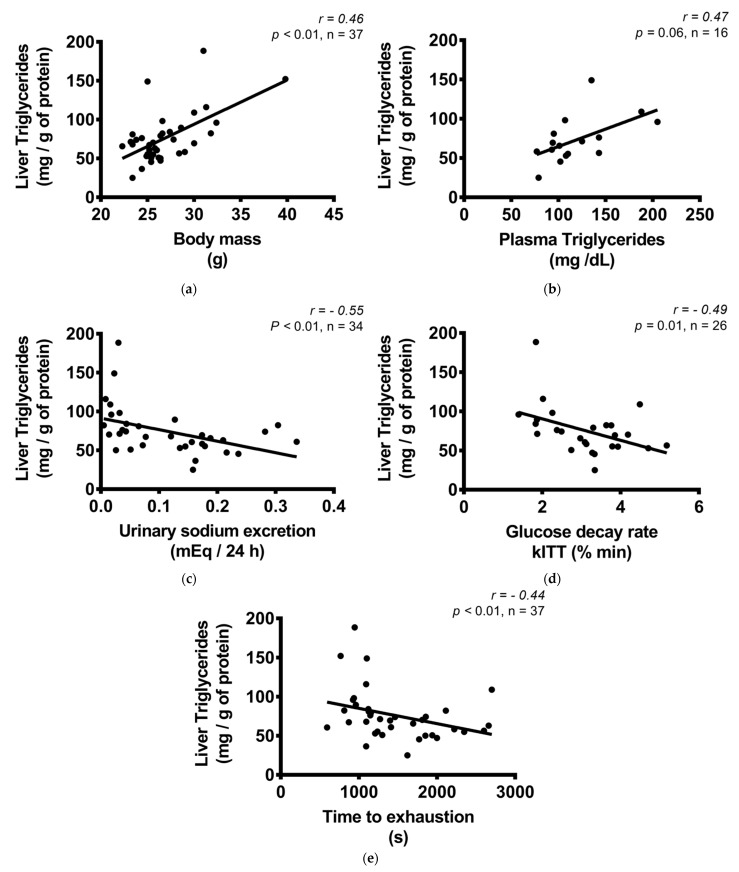
Correlation between liver triglycerides (mg/g of protein) and body mass, biochemical variables, and exercise treadmill test. (**a**) Liver triglycerides (mg/g of protein) and body mass (g); (**b**) liver triglycerides (mg/g of protein) and plasma triglycerides (mg/dL); (**c**) liver triglycerides (mg/g of protein) and urinary sodium excretion (mEq/24 h); (**d**) liver triglycerides (mg/g of protein) and glucose decay rate, kITT (% min); (**e**) liver triglycerides (mg/g of protein) and time to exhaustion in treadmill exercise test (s). Correlations were performed by the Spearman’s rank correlation test.

The hepatic TG content was increased in the LS-S group, but not in the LS-T group (Figure 5a,c). The content of TG was positively correlated with body mass and plasma TG, and negatively with exercise capacity, insulin sensitivity, and urinary sodium excretion (Figure 6a–e). No difference was observed in hepatic glycogen and cholesterol concentration among groups (Figure 5b,d,e). The liver mass was reduced in LS groups (Figure 5f).

### 3.6. Gene Expression

**Figure 7:**
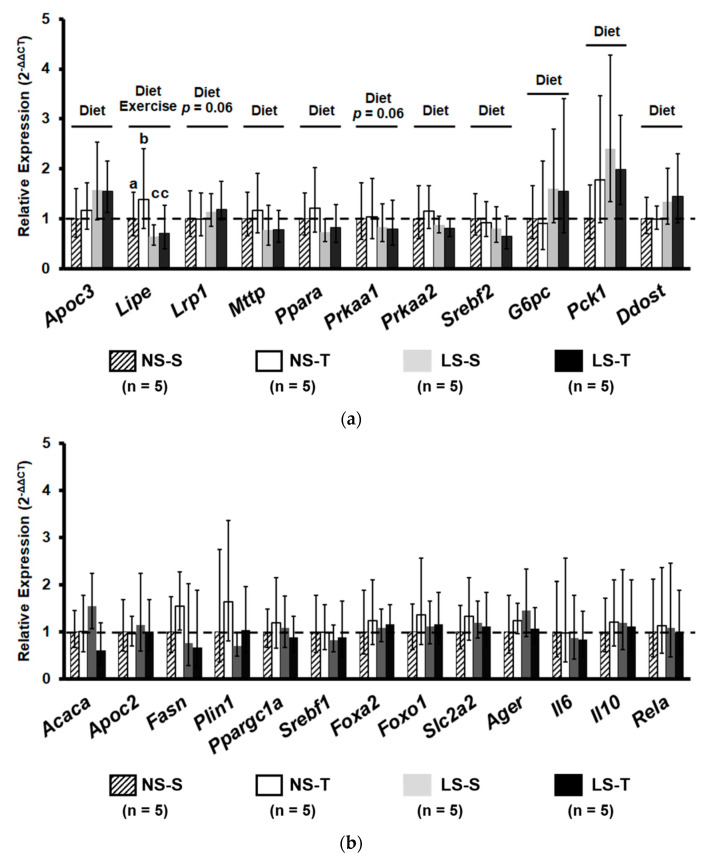
Relative gene expression (2^−ΔΔCT^) of proteins related to lipid and carbohydrate metabolism and inflammation. Gene expression evaluated in liver homogenates. (**a**) Genes with statistical significance between intervention. (**b**) Genes without statistical difference. Data presented as mean ± range. Results compared by 2-way ANOVA (GLM; fixed factor: diet and exercise) with Tukey’s post hoc test. Distinct letters represent statistical differences among groups (*p* < 0.05). “Diet” = *p* < 0.05 NS vs. LS. Exercise = *p* < 0.05 S vs. T. NS = normal-sodium diet; LS = low-sodium diet; S = sedentary; T = trained. *Apoc3*, apolipoprotein c-III; *Lipe*, lipase, hormone sensitive; *Lrp1*, low density lipoprotein receptor-related protein 1; *Mttp*, microsomal triglyceride transfer protein; *Ppara*, peroxisome proliferator activated receptor alpha; *Prkaa1* and 2, protein kinase, AMP-activated, alpha 1 and alpha 2 catalytic subunit; *Srebf1* and *2*, sterol regulatory element binding transcription factor 1 and 2; *G6pc*, glucose-6-phosphatase, catalytic; *Pck1*, phosphoenolpyruvate carboxykinase 1, cytosolic; *Ddost*, dolichyl-di-phosphooligosaccharide-protein glycotransferase; *Acaca*, acetyl-Coenzyme A carboxylase alpha; *Apoc2*, *Apolipoprotein c-II*; *Fasn*, fatty acid synthase; *Plin1*, perilipin 1; *Ppargc1a*, peroxisome proliferative activated receptor, gamma, coactivator 1 alpha; *Foxa2*, forkhead box A2; *Foxo1*, forkhead box O1; *Slc2a2*, solute carrier family 2 (facilitated glucose transporter), member 2; *Ager*, advanced glycosylation end product-specific receptor; *Il6*, Interleukin-6; *Il10*, Interleukin-10; *Rela*, v-rel reticuloendotheliosis viral oncogene homolog A.

The expressions of Apoc3 (that encodes for apoCIII, an inhibitor of lipoprotein lipase, LPL), and Lrp1 (*p* = 0.06; that encodes for LRP-1, the receptor of lipoproteins that contain ApoE) were enhanced by the LS diet (Figure 7a). However, the expression of Ppara and Prkaa1 (*p* = 0.06) and 2 (that encodes for PPARα, AMPKα1 e α2 key proteins in oxidative metabolism), Lipe (that encodes for hormone sensitive lipase, responsible for the hydrolysis of TG), Mttp (that encodes for MTP, important for the VLDL assembly), and Srebf2, (that encodes for SREBP2, a transcription factor that regulates cholesterol homeostasis) were diminished by the LS diet. The AET only increased the expression of Lipe in the NS-T group as compared to the other groups (Figure 7a). Moreover, the LS diet increased the expression of genes related to glucose metabolism, G6pc and Pck1, both enzymes of gluconeogenesis, and the anti-inflammatory and antioxidative gene, Ddost (that encodes AGR1, a receptor that counteracts the oxidative and inflammatory response elicited by glycoxidative stress) (Figure 7a). Other genes involved in lipid metabolism (Acaca, Fasn, ApoC2, Plin1, Ppargc1a, Srebf1), insulin signaling (Foxa2, Foxo1, Scl2a2), and inflammation (Ager, Il6, Il10, Rela) were not altered by diet and AET (Figure 7b).

### 3.7. Citrate Synthase Activity 

**Figure 8:**
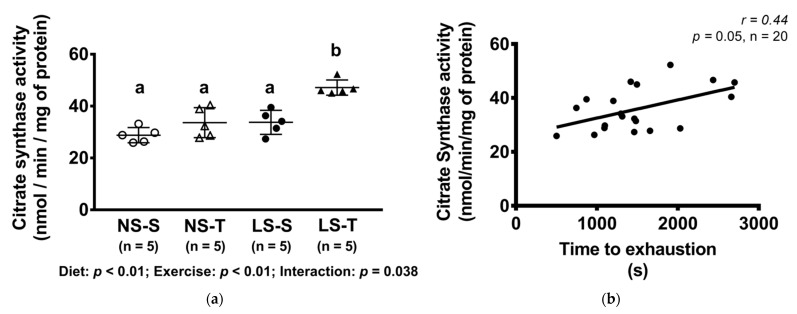
Citrate synthase activity (nmol/min/mg of protein) and correlation between citrate synthase activity (nmol/min/mg of protein) and time to exhaustion in treadmill exercise test. Data presented as mean ± SD. Results compared by 2-way ANOVA (GLM; fixed factor: diet and exercise) with Tukey’s post hoc test. Correlation was performed by Spearman’s rank correlation test (**b**). In (**a**), *p* diet < 0.01; *p* exercise < 0.01; *p* diet*exercise = 0.04. Distinct letters represent statistical differences among groups (*p* < 0.05). NS = normal-sodium diet; LS = low-sodium diet; S = sedentary; T = trained.

The activity of citrate synthase was higher in the LS-T group as compared to other groups (Figure 8a) and it was positively correlated to exercise capacity (Figure 8b). 

## 4. Discussion

In this investigation, it was demonstrated that AET prevented insulin resistance and fat accumulation in the liver of dyslipidemic mice chronically fed a LS diet. Dietary sodium restriction plays a beneficial role in reducing BP and associated comorbidities, although a negative interference in lipid and glucose homeostasis has been described in humans and animal models [3,16,17,18]. In the LDLr-KO dyslipidemic mice, the effects of an intensive dietary sodium restriction in enhancing plasma TG and worsening insulin resistance as firstly demonstrated in rats were confirmed [19]. In those animals, plasma TG enhanced 71% as compared to rats in the NS diet that was attributed to a reduction in VLDL catabolism by the LPL independently of changes in hepatic TG synthesis [19]. 

Insulin modulates the metabolism of TG-rich lipoproteins (namely chylomicrons, VLDL, and intermediate- density lipoprotein, IDL) by inducing the *Lpl* gene transcription, and indirectly by controlling the enzyme activity. This is particularly related to the ability of insulin in increasing and reducing the expression of, respectively, *Apoc2* and *Apoc3* genes in the liver. Conceivably, apoCII favors while apoCIII inhibits the LPL activity. 

Although presenting normal food intake as compared to other experimental groups, the LS diet groups showed increased body mass, a clinical condition that is in many cases accompanied by insulin signaling impairment, hypertriglyceridemia, and liver fat accumulation. In fact, the body mass was one of the main variables associated with hepatic TG. The rise in body mass has been reported by others [20,21] and attributed to changes in energy expenditure [20] rather than increased food consumption, and to the adipogenic effects of angiotensin II [22].

Insulin resistance has previously been reported in animal models and a higher HOMA index was described in humans both submitted to intense dietary sodium restriction [5,18,23]. The insulin-resistant state that is observed in LS diets is thought to be related to the activation of RAAS and the sympathetic nervous system [23]. Moreover, the increased FFA flux to the peripheral insulin-sensitive organs such as adipose tissue and skeletal muscle impairs insulin signaling. In LDLr-KO mice, the peripheral IR elicited by the LS diet was attributed to alterations in the gastrocnemius lipidomics [4]. In the liver, the mechanisms that induce IR are less clear and may include inflammation and lipid accumulation that accompany obesity [24]. In the present investigation, it was demonstrated that dietary sodium restriction altered the expression of insulin-regulated genes that control lipid and carbohydrate metabolism in the liver. Those changes still seem incipient and could be more evident in long-term dietary salt restriction. 

Particularly, an increased expression of *Pck1* and *G6pc* was observed, contributing to the enhanced flux along the gluconeogenic pathway that characterizes hepatic IR states and enhances plasma glucose levels. It has been shown that angiotensin II increases hepatic gluconeogenesis by stimulating glycogen phosphorylase activity [25], which may have favored a hepatic insulin resistance state in the LS-S mice. In addition, the increased mRNA levels of *Apoc3*, which is usually downregulated by insulin, reasserts some degree of IR in the liver contributing to hypertriglyceridemia by the diminished TG hydrolysis by the LPL. On the contrary, the expression of *Mttp* was surprisingly reduced and may have accounted for the accumulation of TG in the liver of LS diet animals. Moreover, hepatic lipid accumulation may have been favored by the reduction in the expression of genes that control oxidative metabolism, such as *Prkaa1* and *2* and *Ppara*, and lipolysis (*Lipe*), and a trend for the increased expression of *Lrp1* (*p* = 0.059) that mediates the hepatic uptake of TG-rich lipoproteins. 

A reduced expression and/or activity of AMPK in kidneys and skeletal muscle of animals submitted to an LS diet or treated with angiotensin II was previously demonstrated [26,27]. Moreover, pharmacological or genetic manipulation of AMPK and PPAR alpha are inversely associated with hepatic TG concentration, since they improve FFA metabolism by beta-oxidation [28]. 

The role of hepatic hormone-sensitive lipase (*Lipe*) in TG lipolysis is not well known. Subjects with hepatic steatosis showed an 80% reduction in hepatic hormone sensitive lipase expression expression [29], and liver-specific *Lipe* overexpression in mice dramatically reduced the concentration of hepatic TG [30]. However, Xia et al. (2017) did not observe changes in hepatic lipid content in mice with deletion of hepatic *Lipe*, suggesting that other lipases may compensate for the hormone sensitive lipase reduction [31]. In the present investigation, the expression *Srebf1* was not changed while *Srebf2* was reduced by the LS diet, although the involvement of this gene is preferentially related to cholesterol synthesis and LDL uptake by the B-E receptor. Hepatic glycogen, another marker of IR, was unaffected by the LS diet and those discrepancies may be related to the length of the experimental protocol and to the degree of hepatic IR that was not determined.

In addition, inflammation and glycoxidative stress elicited by LS diet [3] are related to the development of nonalcoholic steatosis [32]. Interestingly, *Ddost* expression was increased by the LS diet, probably as a compensatory mechanism. Many other genes related to lipid metabolism and insulin signaling were unaltered by diet or AET, which may relate to the degree of IR achieved and by the length of the experimental protocol.

Xavier et al. (2003) investigated the lipogenic effects of the LS diet and found an increased amount of lipids in rat carcasses and retroperitoneal fat pads, but not in the liver despite an increase in hepatic lipogenesis, which may be ascribed to the different animal model utilized by them. Insulin resistance triggers liver steatosis by disturbing the balance among lipid synthesis, clearance, and lipoprotein uptake [6]. 

Changes in lifestyle represent the first line for the treatment of hepatic steatosis [33], and several studies have demonstrated a protective role of exercise training in preventing liver fat accumulation, regardless of body weight loss and even in a low-volume and low-intensity program [34,35,36,37]. The increase in fatty acid oxidation, mitochondrial biogenesis and damage prevention, and reduction of lipogenesis and insulin resistance in the liver and peripheral organs are the major effects of exercise. Accordingly, it was observed in the present investigation that AET reduced insulin resistance optimizing glucose disposal rate in the kITT. Moreover, exercise training reduced intrahepatic TG accumulation, which was associated with increased citrate synthase activity that positively correlated with AET capacity (time to exhaustion test). Nonetheless, the effects of exercise training on gene expression were not evidenced, which may be related to the exercise intensity. The exercise intensity was not adjusted throughout the experimental protocol, and the velocity of running (15 m/min) represented between 56% (at first week) and 39% (at the final) of peak speed in the progressive exercise test, suggesting moderate- to low-intensity physical training.

The present investigation has some limitations. There are no ideal mouse models that fully mimic the particularities of human glycolipid metabolism as well as the pathophysiology of human dyslipidemia, insulin resistance, and metabolic syndrome. Several animal models have been established to investigate the mechanisms of dyslipidemia and insulin resistance, which are some of the main risk factors for cardiovascular disease morbidity and mortality. The selection of an experimental model that enables a satisfactory understanding of the systemic and hepatic effects of dietary sodium restriction is extremely complex, since all animal models show advantages and disadvantages. Considering all of the experimental limitations, the LDLr-KO mice allow the investigation of the dietary sodium restriction effects on hepatic tissue and, simultaneously, reduce the influence of some important interfering factors on the experimental design. They include in wild-type mice the use of a high-fat diet containing cholic acid or a diet high in fats and sucrose that may induce hepatotoxicity and inflammation. Although the obtained results must be confirmed in humans, the use of LDLr-KO mice—a model that mimics the pathophysiology of human dyslipidemia—in the current study, allowed showing important adverse effects of dietary sodium restriction on the liver and their modulation by aerobic exercise training, accentuating the clinical perspective of the present investigation.

People with dyslipidemia and metabolic syndrome are at high risk of non-alcoholic fatty liver disease, hypertension, and cardiovascular disease, which make them important candidates for dietary sodium restriction. Results in dyslipidemic LDLr-KO mice agree with other studies in Wistar rats [6] and wild-type mice [7] that showed increased hepatic lipogenesis and dyslipidemia induced by the LS diet. However, observational data in humans associate sodium restriction with a lower odds ratio for non-alcoholic fatty liver disease [38,39,40]. Considering that dietary sodium intake is also related to other nutritional components and alimentary habits, clinical trials are necessary to translate results from murine models to human health.

Although the salt restriction was quite intense in the experimental protocol utilized, to the best of our knowledge this is the first demonstration that the LS diet alone induces TG accumulation in the liver due to IR and altered gene expression. Liver TG was positively related to body mass and plasma TG levels and inversely correlated with sodium urinary excretion, and glucose decay rate. It is noteworthy that although data obtained in this investigation refer to gene expressions that in some cases do not reflect protein content and/or activity, findings are in line with the biochemical assessment of TG content in the liver and with the assessment of IR by the kITT. 

Importantly, the results evidence that AET is an important tool to mitigate the deleterious effects of the LS diet in lipid and glucose homeostasis avoiding systemic IR and liver steatosis. Exercise performance negatively correlated with the amount of TG in the liver. This is particularly important, considering that the benefits of dietary sodium restriction in reducing BP can be maintained in association with regular exercise and deserves further investigation in humans.

## 5. Conclusions

Intensive dietary sodium restriction in LDL receptor knockout mice increased body mass, triglyceridemia, glycemia, and IR. In the liver, the LS diet favored TG accumulation, which was associated with modulation in the expression of genes related to gluconeogenesis, VLDL uptake and assembly, lipolysis, and oxidative metabolism. Aerobic exercise training prevented lipid accumulation in the liver due to its protective role in stimulating hepatic oxidative metabolism and reducing peripheral insulin resistance.

## Figures and Tables

**Table 1:** Characterization of groups before intervention.

	NS-S (*n* = 12–14)	NS-T (*n* = 13–11)	LS-S (*n* = 13–11)	LS-T (*n* = 13–10)	*p*
Body Mass (g)	24 ± 2	25 ± 1	25 ± 2	24 ± 2	0.76
TC (mg/dL)	268 ± 36	255 ± 37	288 ± 34	265 ± 40	0.15
TG (mg/dL)	168 ± 36	150 ± 24	157 ± 32	152 ± 26	0.37
Glucose (mg/dL)	93 ± 12	93 ± 8	93 ± 14	95 ± 14	0.93
Hematocrit (%)	49 ± 5	49 ± 7	49 ± 7	51 ± 5	0.87
SBP (mmHg)	109 ± 8	113 ± 6	111 ± 6	111 ± 5	0.44
DBP (mmHg)	47 ± 8	44 ± 7	46 ± 14	48 ± 9	0.85
HR (bpm)	522 ± 75	481 ± 55	484 ± 53	475 ± 82	0.39

Data presented as mean ± SD. Results compared by one-way ANOVA. NS = normal-sodium diet; LS = low-sodium diet; S = sedentary; T = trained; TC = total cholesterol; TG = triglycerides; SBP = systolic blood pressure; DBP = diastolic blood pressure; HR = heart rate.

**Table 2:** Characterization of groups after intervention.

	NS-S (*n* = 14–12)	NS-T (*n* = 13–11)	LS-S (*n* = 13–11)	LS-T (*n* = 13–11)	*p*
Body Mass (g)	26 ± 3	26 ± 2	29 ± 5	27 ± 2	Diet
TC * (mg/dL)	271 ± 103	304 ± 91	254 ± 96	292 ± 83	-
TG * (mg/dL)	101 ± 9	100 ± 27	152 ± 36	130 ± 51	Diet
Glucose (mg/dL)	100 ± 15	115 ± 20	115 ± 20	124 ± 22	Diet Exercise
Hematocrit (%)	48 ± 3	50 ± 3	50 ± 3	51 ± 5	-
SBP (mmHg)	106 ± 6	106 ± 6	105 ± 8	102 ± 5	-
DBP (mmHg)	46 ± 8	49 ± 14	44 ± 9	46 ± 11	-
HR (bpm)	531 ± 60	531 ± 46	535 ± 61	530 ± 63	-

Data presented as mean ± SD. Results compared by 2-way ANOVA (GLM; fixed factor: diet and exercise). “Diet” = *p* < 0.05 for NS vs. LS; “Exercise” = *p* < 0.05 for S vs. T. NS = normal-sodium diet; LS = low-sodium diet; S = sedentary; T = trained; TC = total cholesterol; TG = triglycerides; SBP = systolic blood pressure; DBP = diastolic blood pressure; HR = heart rate. * (*n* = 3–5).

## Data Availability

All data reported are included in the manuscript and upon personal request to the authors raw data can be kindly shared. This article includes original data and only kITT and biochemical data of the LS and NS groups were shared with another previous publication as informed.
